# Some Cases of Peripheral Neuritis

**Published:** 1890-12

**Authors:** D. R. Paterson

**Affiliations:** Cardiff


### Clinical Records.

---

### SOME CASES OF PERIPHERAL NEURITIS.

By D. R. Paterson, M.D. (Edin.), M.R.C.P. (Lond.), Cardiff.

Although the great Irish physician, Graves, was many years ago conversant with the fact that paralysis might arise from an affection of the "nervous extremities alone," it is only within recent times that the observations of Leyden, Grainger Stewart, and others have placed it upon an established basis. The clinical picture of peripheral or multiple neuritis is, indeed, now so well known, that its recognition presents but little difficulty; and our knowledge of the conditions under which we may expect to find it is ever increasing. Several points of interest, however, connected with the disorder are still *sub judice*, and call for settlement; and as I have had opportunities of studying several examples lately, I may be permitted, in illustration, to detail some of the clinical histories, condensed from notes which I have made. I have to acknowledge my indebtedness to Dr. Sheen, under whose care, at the Cardiff Union Hospital, most of the patients were.

Case I.—*Multiple Neuritis affecting arms and legs—Phthisis.*—K. R., 23, a single woman of loose character, was admitted April 11th, 1890, complaining of "rheumatism." She stated she had been in the hospital for six weeks at the end of last year, on account of pain in the feet and inability to walk properly. She improved considerably, and got about so well as to take her discharge before Christmas. She resumed her drinking habits, and in a month was laid up with the old symptoms, being obliged to stay in bed, as she was unable to stand. A month before admission the fingers began to feel dead, with pain shooting down into them and the wrists, and weakness and difficulty of grasping anything appeared. She had suffered from cough for some time, certainly before last Christmas, but had no expectoration until a few weeks before admission; and latterly she had been losing flesh rapidly. For the last three years she had drunk heavily, chiefly spirits. There was no definite history of syphilis. *On admission* the patient showed signs of emaciation. There was decided weakness of the hands, both in the movement of the fingers and grasping power. The movements were slow and clumsy, the patient being able to pick up a pin imperfectly, and the grasp of the right hand was stronger than that of the left. Sensation was impaired in the fingers and backs of both hands; but in some places, *e.g.* the palms, there was hyperæsthesia. In the lower extremities, the patient had pain in the thighs and calves, very severe on moving the limbs, and great tenderness on grasping the muscles. There was much wasting. The extent to which she could lift both legs from the bed was small, and the power of dorsal-flexion in the ankle feeble, the toes being "pointed." She was unable to stand without support; the plantar reflex was lively, but both knee-jerks absent. Sensibility was impaired, more especially over the calves, while above the knees the hyperæsthesia was so great that mere stroking caused great agony. The sensory disturbances went up as far as Poupart's ligament. There was full control over rectum and bladder. Frequently, palpitation on exertion troubled her. She was suffering from diarrhœa; the cough was troublesome, and the expectoration, although scanty, muco-purulent. There was impaired resonance at the right apex, with cavernous breathing; increased vocal fremitus and vocal resonance, and crepitation. At the left apex the signs were not advanced. The temperature ranged between 100° and 103°, and night-sweats were frequent. On May 9th, dulness had become more marked at the right apex; and patient was still in bed, on account of the great tenderness in the thighs and the continuance of diarrhœa. By June 4th there was, on the whole, less pain, but a good deal of tenderness in calves and thighs; wasting becoming more prominent and power of movement less, the legs being extended and the toes pointed. Sensation altered in the upper limbs as far as the middle of the upper arm. Left apex more affected. On the 10th there was a severe exacerbation of the pains, ascribed to the wet weather; and sensation below the knee was now quite gone. Diarrhœa was checked after bismuth and hydrocyanic acid mixture. A month later (July 7th) pain was considerable, "burning like a coal," from the toes to the hips. The arms had been free from pain for some time. Urine contained a trace of albumen. Death took place three days after; and at the autopsy a cavity as large as a Tangerine orange was found in the right upper lobe, with irregular walls and much consolidation around. The left upper lobe was solid, and scattered foci were seen throughout the entire lung. No naked eye changes were observed in several of the peripheral nerves removed for microscopic examination.

CASE II.—*Acute Phthisis—Peripheral Neuritis affecting the legs.*—S. H., 22, who had "drunk hard during last five years," was admitted on December 30th, 1889, with acute lung disease of three weeks' standing. It had begun with shivering; and she continued to go about drinking until the week before admission, when she took to bed. On admission there was consolidation at the left apex, and moist sounds; while behind and below there were loud râles. The temperature was 102°; and the sputum was muco-purulent, with numerous tubercle bacilli. On January 4th the patient first complained of "burning, aching pain" just above the knees, sometimes extending down to the feet and up to the hips. By the 6th the symptoms of peripheral neuritis were pronounced. It was painful to move the limbs, and she was unable to stand. The legs felt "dead;" and whilst tactile sensation was retained, the sensibility to pain was much blunted from Poupart's ligament down. Both plantar reflexes and knee-jerks were absent, and the calves and thighs tender to touch. On the 15th she was unable to turn in bed. By the 18th the pain had lessened, the limbs lay extended, toes pointed and feet everted, with the power of extension and flexion in the ankle slight, and the movements of the knee feeble. There was dulness over the whole upper lobe of the left lung, bronchial breathing, increased vocal resonance and crepitation; while over the lower half, and the upper half of the right, there was crepitation without impaired resonance. All other organs were healthy, the urine normal, and the temperature 102°. Later on, while pain disappeared and the leg-muscles became much wasted, a cavity formed in the middle of the left lung, night-sweats and diarrhœa became more continuous, and death ensued on February 6th; the temperature having ranged in the course of the illness between 100° and 103°. On *post mortem* examination, the left lung was found solid with tuberculous matter, and a small cavity in the upper lobe ; extreme apex of right lung also involved. The sciatic and peroneal nerves of both legs were swollen and injected, the left being more so.

Here the neuritis began while the patient was under observation, and a week after all alcohol was stopped. It will be observed that it was limited to the lower limbs.

CASE III.—*Phthisis—Neuritis affecting legs and arms—Mental Symptoms.*—E. S., aged 36, a married woman ; admitted September 11th. She had drunk heavily for a long time, and within recent years was on several occasions in hospital suffering from the effects of drink. She complained of cough and weakness for about a year, and five months before admission had severe hæmoptysis. Three months after, stiffness and pain in the legs appeared ; in two or three weeks she was obliged to sit down from the difficulty of walking and pain in the soles, ankles and legs generally, and within a month from the commencement was unable to walk. Shortly after the involvement of the legs, she experienced loss of power and numbness in the hands, and this condition increased. Besides expectorating much muco-purulent sputa, and showing signs of extensive affection of the left lung, the patient presented the appearances of peripheral neuritis in an advanced stage, and very similar to those described in Case I. On October 28th, a few days before death, there was little alteration in the legs. But she had delusions about her surroundings : she did not know how long she had been in hospital, and forgot when she saw her friends last. She was not noisy, and she slept well during the daytime. These continued up to the end.

CASE IV.—*Phthisis—Neuritis of legs.*—E. T., 35, a charwoman of dissipated habits. Early in this year she had been in hospital with an injury to the knee-joint, and went out well before Whitsuntide. She continued in good health for two months, although she lay out at nights and drank hard of spirits, rum, etc. Cough, however, came on, and hæmoptysis, and shortly before admission—at the end of August—she began to have severe pains in the legs, and soon lost the power of walking. On examination, there a moderate degree of paralysis of the legs with altered sensation, tenderness of the muscles and absence of knee-jerks. In the right hand, a feeling of formication. The right lung was largely affected, cavernous respiration could be heard, and the sputum was copious and muco-purulent. The state of the limbs improved after admission, but the pulmonary disease advanced, and the patient died in November.

CASE V.—*Phthisis—Multiple Neuritis—Mental Symptoms.*—A lady of 28, five years married, but childless, came under the care of a medical man in this town in October, 1889, when a phthisical cavity at the left apex was recognised. Under treatment the general condition was improving, when a little before Christmas the patient began to complain of curious sensations, pain in walking and tenderness of the legs as far as the hips. She was unable to sleep on account of cramps, and was obliged to lay up. Pain became more severe and burning, patches of anæsthesia appeared on the sole and heel, and the hyperæsthesia of the legs and knees increased, so that the patient dreaded anything touching her. Soon after tingling of the finger-tips set in, followed by pain and loss of power, rendering it impossible for her to feed herself or hold a glass. The leg-muscles wasted, the toes became pointed, although the temperature at this time was little above normal. Later on, muscular twitchings in the face, arms, and legs were observed, and lasted a few days; after which there was a partial convulsion, which left for some days an external squint and double vision. Mental symptoms, such as hallucinations and delusions, with mumbling speech, were likewise present. After some days the patient improved mentally, and regained some power over the legs; but the whole body remained hyperæsthetic. She died about three months after the onset of the neuritis, the lung symptoms never being very prominent. There was a suspicion of alcohol, which, however, was always denied, until the patient became quite bedridden and mental symptoms appeared, when the friends admitted that she was in the habit of consuming considerable quantities of wines and spirits daily. The improvement followed its restriction.

In these cases we have a striking example of the fact that the female sex is especially liable to alcoholic neuritis, and further an illustration of the association of that disease with tuberculosis. We observe that the patient in each instance was affected with phthisis; and, with the doubtful exception of the last (Case V.), death was to be attributed to that cause. In the discussion on chronic alcoholism at the Pathological Society two years ago, Dr. Payne called attention to the "undoubted frequency of tubercular disease in the subjects of alcoholic paralysis"; and Dr. Sharkey threw out the suggestion that a suitable soil for tubercle might result from a neuritis of branches of the vagus or other nerve going to the lungs. Such an explanation may serve in some instances, and I can recall the case of a seaman, aged 43, suffering from phthisis, whose latter days were rendered miserable by frequent attacks of distressing palpitation, and in whom, after death, besides extensive infiltration of both lungs, the vagi were found surrounded by tuberculous glands, being softer, redder, and more flattened than usual. Even here it seems probable that the changes in the nerve were secondary. However that may be, it is to be noticed that in the cases I have recorded above, the tuberculous process was first upon the scene. In each patient symptoms or signs of phthisis had shown themselves some time before the nerve affection, and it is difficult to look upon the former as a complication of the latter, or caused by it. On the contrary, these cases suggest tubercle as the chief factor, and such a form of neuritis has indeed been described by Pitres and Vaillard; but in nearly all the cases they record there is a history of alcohol as well. The part played by alcohol was well exemplified in Case I. Whilst it is probable that phthisis as well as neuritis was present on the patient's first admission, the latter rapidly improved as soon as all stimulants were cut off, only to reappear, however, in a more aggravated form when she returned to her old habits. Dr. Pitt, at the Pathological Society meeting, gave some striking statistics of the association of alcoholism and tubercle. In 1883, of eight cases of cirrhosis of the liver observed in the *post mortem* room at Guy's, acute tuberculous disease was found in six, and fibroid phthisis in one of the other two. This, he says, is probably exceptional. But frequently the pulmonary symptoms have not been prominent, and therefore may be overlooked. In all the patients under 40 who had cirrhosis, out of thirty-three two-thirds had tubercle in the lungs or elsewhere. To state the matter generally, we may say that the causal relation of tuberculosis and alcoholism, is to be explained on the principle that drink, with the conditions too often attendant upon it, impairs the general vitality and renders the individual, with his enfeebled energy, but little able to resist the onset of disease. These two processes intimately react upon each other, the order in which they appear being probably determined by circumstances peculiar to the individual. Moreover, we have often to reckon with the possibility of syphilitic infection; and here I may allude to a point lately raised, as it furnishes a good example of the difficulty of determining the ætiological factor. Cases of syphilitic neuritis have been repeatedly described; but in many of them we cannot altogether exclude the effects of alcohol; nor, what is of more importance, ought we to lose sight of the influence of the mercury used in treatment. It is well known that most of the heavy metals act as powerful nerve poisons, and two at least—lead and, less frequently, arsenic—are recognised causes of polyneuritis. In the old days of water-gilding, the picture of tremor and other nerve disturbance dependent on mercurial vapour was not uncommon; and it seems feasible that a prolonged course of inunction may at length affect the patient's nerves and illustrate what Lord Palmerston said regarding gout, about the remedy being worse than the disease. Accordingly, the question coming up for decision would be, as happened in an instance quoted by Oppenheim, Does the neuritis depend upon the syphilitic poison or arise from the mercurial treatment? The importance of this is apparent, and was amply shown by Oppenheim's experience; since, on discontinuing the energetic mercurial treatment under which the disease progressed, and substituting baths, massage, etc., the best possible results were obtained. Syphilis and alcohol are frequently companions, and it is often impossible to assign their priority in causation. Thus, to quote an allied case, I saw not long ago a well-marked example of Charcot's amyotrophic lateral sclerosis in a man who, whilst admitting that he had syphilis thirty years before, attributed his illness to six months' hard drinking. But it is open to question whether either had any influence; at all events, treatment met with no success. Quite recently Dr. Buzzard published some observations which go to show that the action of alcohol may be circumscribed, and he quotes an instance of its limitation to a single muscle—the extensor communis digitorum. I do not know how far the writer was able to exclude local causes; but some limited paralyses in drunkards, we are still justified in grouping amongst those regarded by Duchenne as "paralysis from cold." I have notes of the case of a labourer, aged 51, who, whilst working last winter through a sleety night shovelling coal, experienced towards morning some stiffness in the right forearm, and on warming it at the fire, felt it "go quite dead." The hand became powerless; and when I saw him, two weeks after the onset, there was pronounced "drop-wrist," paralysis, and wasting of the muscles supplied by the musculo-spiral nerve, and impaired sensation in its cutaneous area, with numbness and a feeling of "pins and needles." No traces of lead were present; but the patient admitted being a drinker. Recovery was complete in ten weeks.

Turning now from this part of the subject, I should like to refer briefly to a problem which is at present engaging discussion. I allude to the association of abnormal mental states with this disease. In Cases III. and V. mental symptoms appeared at a late stage, and may possibly have little significance; but where the mind is early impaired, they form such a prominent feature, that the neuritis may be lost sight of altogether, or mistaken for another affection. The following history illustrates this:

CASE VI.—*Peripheral Neuritis—Mental Symptoms.*—C. B., aged 22, admitted 23rd April, 1889, with certificate that she was suffering from locomotor ataxy. No history could be got from her friends, beyond that she had been "very bad" for some weeks, and previous to that had been drinking heavily. She lay on her side and scarcely moved. Her mind was a perfect blank. She was unable to give any account of herself; did not know where she was, or how long she had been in the hospital; and when asked anything, stared stupidly, and said "I forget," or "I don't know." There was inability to stand, the leg movements were very feeble, the power of dorsal-flexing the ankle gone, the muscles wasted, and both calves and the left thigh tender. Sensibility below the knee was lost. The arms were weak, the grasp feeble, and both biceps muscles tender; but sensation was good. The knee-jerks were absent; the pupils reacted to light and accommodation; and the tongue was tremulous. After admission, she began to improve under massage and baths: intelligence returned; the face regained expression; and she was able to walk with only slight hesitation on her discharge three months later. She could not recall, during her stay, anything of the early part of her illness; memory, however, was slowly becoming better before she left.

Here the symptoms were sufficiently marked to give some colour to a suspicion that we had to deal with a case of mental disease. The hebetude, expressionless face, and complete loss of memory, suggested an illness of longer duration than was indicated by the meagre history; but those doubts were quickly dispelled by the patient's rapid progress, and we were convinced of the truth of the opinion hazarded on the patient's admission by one of her friends, that it was "nothing but the drink." The psychical disturbances as they accompany neuritis have been fully studied in recent times by Korsakow, who has pointed out their relation to alcoholism, and proposes to separate them into a special group. Be that as it may, the practical point urged by Oppenheim is, that such a condition may be mistaken—and this actually occurred in the case he quotes, as well as in my own—for tabes with mental symptoms, wherein, of course, the prognosis is altogether different.

Among the acute infectious diseases in which peripheral neuritis may be met with is enteric fever. The subjoined case is of some interest.

CASE VII.—*Enteric Fever—Peripheral Neuritis affecting legs.*—H. M., 31, a German sailor, was admitted April 21st, 1890, with enteric fever in the third week, and severe pulmonary congestion. On the following day there was a small intestinal hæmorrhage. The temperature became normal on the 28th; this was followed by a slight relapse, the temperature again reaching 98.4° on May 7th. Next day the patient complained of pain in both legs, and the temperature rose to 100.6°. On the 9th he was much distressed at the state of his legs. They lay extended, with the feet inclined outwards, there being inability to use them beyond slight flexion of the knees and ankles and feeble movement of the toes. The calves were very tender to the touch, and movement of the ankles caused pain in them. Sensation was good, the plantar reflexes intact, and the joints normal. The arms, patient said, felt weaker; the grasp was feeble, there was pain in the flexor muscles, and pressure on the ulnar nerve showed tenderness. On the 12th, after massage, the legs could be moved better, and the tenderness was less. By the 20th power was being regained rapidly. He was discharged on the 28th, at his own request, with some weakness remaining in the peronei.

The sudden onset of loss of power in the legs, and the absence of sensory disturbance, resemble the palsy occasionally seen after diphtheria. I do not know that the nerves of the upper limbs could be said to be the seat of neuritis: the patient attributed the weakness of his arms to the extra work thrown upon them in consequence of the state of the legs,—movements such as turning, sitting up, &c., necessitating much assistance from the use of the hands,—and it is likewise probable that the tenderness of the ulnar nerve did not denote more than is usually met with in convalescence from acute disease. In pneumonia, during convalescence, I have observed, in two instances, similar symptoms confined to the leg, though not so pronounced. Paresis and muscular tenderness were made out, but sensation was intact.

